# Giant honey bee (*Apis dorsata*) sting and acute limb ischemia: a case report and review of the literature

**DOI:** 10.1186/s13104-018-3422-6

**Published:** 2018-05-21

**Authors:** Gowri M. Ratnayake, P. N. Weerathunga, Matharage Shani Apsara Dilrukshi, E. W. R. Amara Witharana, Saroj Jayasinghe

**Affiliations:** 1 National Hospital of Sri Lanka, Colombo 10, Sri Lanka; 2 Base Hospital Deniyaya, Deniyaya, Sri Lanka; 3 Department of Clinical Medicine, University of Colombo, Colombo 8, Sri Lanka

**Keywords:** Bee sting, Acute limb ischaemia

## Abstract

**Background:**

Clinically significant manifestations of Hymenopteran envenomation is increasingly recognized in Sri Lanka. These clinical manifestations range from localized allergic reactions to end-organ failure and thrombotic-episodes. We report a case of 65 year old male who developed acute lower limb ischaemia after a sting of the hymenopteran *Apis dorsata.*

**Case presentation:**

A 65 year old male with hypertension and hyperlipidaemia presented with envenomation from an attack of a swarm of *A. dorsata*. He subsequently developed acute limb ischaemia following an acute femoral thrombus and made a complete recovery with anticoagulation and surgical-embolectomy.

**Conclusions:**

This case adds to the spectrum of thrombotic manifestations of Hymenopteran venom highlighting the requirement for close monitoring and clinical vigilance in these patients.

## Background

Hymenopterans are broadly categorized into three-families; Apidae (honeybees, bumblebees) Vespidae (hornets, wasps, and yellow jackets) and Formicidae (fire ants) [1].

Three-species of honey bees have been reported in Sri Lanka. Among them the giant honey bee (*Apis dorsata*) is the commonest offending Hymenopteran accounting to hospital admission in Sri Lanka. A prior study in Sri Lanka confirmed that 90.7% of Hymenoptera stings were due to the Giant honey bee [1].

*Apis dorsata* sting can cause a spectrum of clinical manifestations ranging from local allergic reactions to mass envenomation and end-organ damage. There are reports of acute kidney injury, myocardial infarction, severe anaphylaxis and bowel gangrene after giant honeybee or wasp sting in Sri Lanka [2–5]. Acute lower limb ischaemia due to possible vasoconstriction and multi-organ failure following wasp sting in Sri Lanka is also reported [6].

We report a case of 65 year old male who developed acute lower limb ischaemia after *A. dorsata* sting. To the best of our knowledge, this is the first reported case published for isolated lower limb ischemia following giant honey bee sting in Sri Lanka.

## Case presentation

A 65 year old male famer from Deniyaya, Sri Lanka was stung by a swarm of 75–100 honey bees while working in his tea plantation. The offending insect was identified as *A. dorsata*. He was a nonsmoker but a patient with hypertension and ischaemic heart disease who was on aspirin 150 mg nocte, atorvastatin 20 mg nocte and losartan 25 mg bd. He was admitted within 1 h of the incident to the local hospital with itching and a generalized erythematous rash. On admission, the patient was hemodynamically stable with a blood pressure of 140/90 mmHg and regular good volume pulse with a rate of 92 beats/min. He was administered Intravenous Hydrocortisone 200 mg, Chlorpheniramine 10 mg. He subsequently developed pain and numbness of his left sided lower limb around 4 p.m. on the same day of admission. Pain and numbness increased over a few hours from admission and his dorsalis pedis pulse became impalpable with a cold non-oedematous lower limb. A clinical diagnosis of acute limb ischaemia was made and intravenous unfractionated heparin infusion was commenced and was subsequently transferred to the National Hospital of Sri Lanka.

On admission to the NHSL the patient was conscious with normal blood pressure, regular good volume pulse and lungs were clear on auscultation with a cyanosed left big and 2nd toe and a cold left lower limb with impalpable dorsalis pedis, posterior tibial pulse. All other peripheral pulses were normal.

He underwent immediate left sided femoral embolectomy under local-anaesthesia. An embolus measuring 7 × 7 × 3 mm was extracted from the femoral artery using a Fogarty catheter and sent for histopathological analysis. Analysis of the extracts confirmed a sterile fresh thrombus with an inflammatory infiltrate. His basic blood investigations are summarized in Table 1. Raised white blood cell count attributable to the systemic reaction caused by the venom. All other blood investigations were normal. His ECG was in sinus rhythm with a heart rate of 88 beats/min. 2D Echocardiogram was normal without a cardiac source of thrombo-embolism. Magnetic Resonance Aortogram (MRA) done post procedure was normal with patent left femoral artery. Intravenous Heparin infusion was continued for another 13 days. He regained his pulses of left lower limb and made and uneventful recovery. He was discharged from the hospital 14 days after admission. The thrombophilia screen was negative.

**Table 1:** Laboratory results

Parameter	Patient’s values	Normal range
White blood cell count (/μl)	13,950	4000–11,000
Haemoglobin (g/dl)	13.8	11–16
Platelet (/μl)	200,000	150,000–400,000
Prothrombin time (s)	13.5	13.5
INR	1.0	
Activated partial thromboplastin time (before heparin) (s)	30.6	24–37
Serum creatinine (μmol/l)	111	60–120
Serum sodium (mmol/l)	139	135–148
Serum potassium (mmol/l)	4.5	3.5–5.1
Fasting blood sugar (mmol/l)	5.1	< 6
Total cholesterol (mmol/l)	4.9	< 5.1
Aspartate transaminase (u/l)	35	10–35
Alanine transaminase	37	10–40 u/l
Total bilirubin (μmol/l)	21	4–21
Total protein (g/l)	65	61–77
Albumin (g/l)	39	36–48
Globulin (g/l)	26	22–40

## Discussion and conclusions

This is a case of acute limb ischaemia following *A. dorsata* sting with a fresh thrombus as evidenced by a leukocyte infiltrate on histopathological analysis. Similar thrombotic events including coronary angiogram proven acute myocardial infarction and bowel gangrene [5] were reported as sequelae of *A. dorsata* envenomation.

*Apis dorsata* venom contains pharmacologically active constituents including histamine, dopamine, noradrenaline, mellitine peptide, hyaluronidase, phospholipase A2 and B, which have vasoactive and thrombogenic effects [7]. These factors induce a pro-coagulant state which may lead to thrombotic events following *A. dorsata* sting.

Pre-existing atherosclerotic vascular system of the lower limb was excluded with a normal lower limb MRA. The possibility of a pre-existing thrombophilic state was excluded with a formal thrombophilia screen. It is interesting that the thrombotic properties of the venom were potent enough to over-ride the antiplatelet effects of long-term aspirin.

This case highlights the requirement for close clinical monitoring of patients with *A. dorsata* sting and high vigilance for thrombotic events especially in rural, resource limited settings.

In conclusion *A. dorsata* stung can lead to variable degree of thrombotic events which can be life and or limb threatening. These complications should be identified and treated promptly to prevent loss of limbs and to reduce mortality. Therefore it is of extreme importance that the practicing clinicians to be aware about thrombotic tendencies associated with *A. dorsata* stung to diagnose and treat promptly.

## Abbreviations

MRA: Magnetic Resonant Aortogram
2-D: 2 dimensional
